# Influence of cement-augmented pedicle screws with different volumes of polymethylmethacrylate in osteoporotic lumbar vertebrae over the adjacent segments: a 3D finite element analysis

**DOI:** 10.1186/s12891-020-03498-6

**Published:** 2020-07-13

**Authors:** Hui-zhi Guo, Shun-cong Zhang, Dan-qing Guo, Yan-huai Ma, Kai Yuan, Yong-xian Li, Jian-cheng Peng, Jing-lan Li, De Liang, Yong-chao Tang

**Affiliations:** 1 The 1st Institute of Clinical Medicine, Guangzhou University of Chinese Medicine, 12 Airport Road, Baiyun District, Guangzhou, 510407 Guangdong People’s Republic of China; 2 Spine Surgery Department, The First Affiliated Hospital of Guangzhou University of Chinese Medicine, Guangzhou, 510407 China

**Keywords:** Cement-augmented pedicle screws, Adjacent segment degeneration, Polymethylmethacrylate, Finite element analysis

## Abstract

**Background:**

Polymethylmethacrylate (PMMA) is commonly used for cement-augmented pedicle screw instrumentation (CAPSI) to improve the fixation stability and reduce the risk of screw loosening in the osteoporotic thoracolumbar spine. Biomechanical researches have shown that various dose of cement (1-3 ml) can be injected to enhance screw stability. To date, there have been no studies on the relationship between adjacent segment degeneration and the volume of PMMA. This study aimed to explore the influence of CAPSI with different volumes of PMMA in osteoporotic lumbar vertebrae over adjacent segments by using finite element analysis.

**Methods:**

Seven different finite element models were reconstructed and simulated under different loading conditions, including (1) an intact model, (2) three single-level CAPSI models with different volumes of PMMA (1, 1.73, and 2.5 ml), and (3) three double-level CAPSI models with different volumes of PMMA (1, 1.73, and 2.5 ml). To improve the accuracy of the finite element analysis, the models of the injectable pedicle screw and bone cement were created by using a three-dimensional scanning machine and the CAPSI patient’s CT data, respectively. The range of motion (ROM), the stress of intervertebral discs, and the stress of facet in the adjacent segment were comparatively analyzed among the different models.

**Results:**

The ROMs of the different segments were compared with experimental data, with good agreement under the different load conditions (21.3°, 13.55°, 13.99°, and 6.11° in flexion, extension, bending, and rotation at L3-S1 level, respectively). Compared with the intact model, the ROM, disc stresses, and facet stress in adjacent segments were found to be higher in the six operative models. Otherwise, with a larger volume of PMMA injected, the ROM, disc stresses, and facet stress slightly increased at the adjacent segment. However, the differences were insignificant with the biggest difference less than 3.8%.

**Conclusions:**

CAPSI could increase the incidence of disk degeneration in the adjacent segment, while within a certain range, different volumes of PMMA provided an approximate impact over the adjacent segment degeneration.

## Background

Posterior lumbar interbody fusion, the classic surgical procedure to treat lumbar degenerative diseases and thoracolumbar fracture, has been developed for more than 70 years since the initial description in 1944 by Briggs and Milligan [1]. A large number of clinical studies have indicated that posterior-approach fusion and fixation can effectively restore sagittal alignment, achieve immediate postoperative stability, and facilitate fusion rates [2–4]. However, consensus holds that the stiffness of the instrument relates directly to increased stress on the adjacent disc and facet joints, which could trigger segmental hypermobility and accelerate adjacent segment degeneration (ASD) [5, 6].

ASD is one of the most common sequelae of spinal interbody fusion and affects the patient’s long-term results. It is usually considered that radiographic degeneration and/or symptomatic degeneration occurs in the upper or lower adjacent segment. The annual incidence of ASD was reported to be approximately 9.8–86.1% in the literature [7, 8]. Studies have shown that advancing age is an independent risk factor for ASD and that aging patients have more obvious degenerative discs, with associated symptoms. In addition, cement-augmented pedicle screw instrumentation (CAPSI) has often been used in elderly people with osteoporosis to increase the pullout strength of the interface between pedicle screws and cancellous bone. None of the previous studies have dealt with biomechanical comparisons of different volumes of polymethylmethacrylate (PMMA) used in osteoporotic lumbar vertebrae over adjacent segments.

The aim of this study was to develop a non-linear finite element (FE) model capable of simulating osteoporotic and fused lumbar spine biomechanics. The purpose is to describe how the PMMA volume used in single- or double-level fixation alters the adjacent discs behavior. Seven different finite element models were generated to compare the range of motion (ROM) and the stress of the intervertebral disc in the adjacent segment, including (1) an intact model, (2) three single-level CAPSI models with different volumes of PMMA (1, 1.73, and 2.5 ml), and (3) three double-level CAPSI models with different volumes of PMMA (1, 1.73, and 2.5 ml).

## Methods

### Development of the intact lumbosacral model

In this study, a healthy adult female volunteer without any history of spinal diseases was selected and the data of her CT scans (AQUIRRON 64, Toshiba, Japan, 250 mAs, 120 kV voltage, slice thickness of 0.625 mm) was obtained from the department of radiology of our hospital. The computed tomography scan images were stored in Digital Imaging and Communications in the Medicine (DICOM) format.

Anatomical 3D models of the lower lumbar vertebrae, sacrum, and coccyx were generated using Mimics research 19.0 (Materialize, Leuven, Belgium). Subsequently, the rough spinal model was imported into Geomagic Studio 2013 (3D Systems Corporation, South Carolina, USA) for further operation, including delete the spikes and the features, making triangles more uniform in size, and generate the surface model. The smoothed model was processed using SolidWorks 2017CAD (SolidWorks Corporation, Concord, MA, USA). Cortical bone, cancellous bone, nucleus pulposus, annulus fibrosus, facet cartilage, and vertebral endplates parts were constructed subsequently. The nucleus pulposus, simulated as a fluid-like and incompressible material, occupied 44% of the disc volume [9]. The thickness of the cortical bone was approximately 0.5 mm [9], and the cartilaginous endplates were modeled to be approximately 1 mm thick [10, 11]. The initial gap between the articulating surfaces was based on computed tomography images and was approximately 0.3–0.6 mm. The above parts were assembled into an intact lumbosacral model.

### Three-dimensional scanning models of pedicle screw

The 3D scanner (Solutionix Rexcan CS+ 3D scanner, SolutioniX, Korea) was applied to scan and build the model of the pedicle screw. The instrument used image registration and 3D matching technology to create a point cloud of the geometric surface by a surface scanning of the target object. Then, the geometric model is generated automatically by the software. Steps to reconstructed the model were as follows: first, the surface of the fenestrated pedicle screw was sprayed with the developer evenly; after that, Ezscan 2017 software was applied to scan the fixed screws automatically; after finishing the scanning, the redundant parts of the 3D models were deleted using the lasso tool, and the file was saved in STL format.

**Fig. 1:**
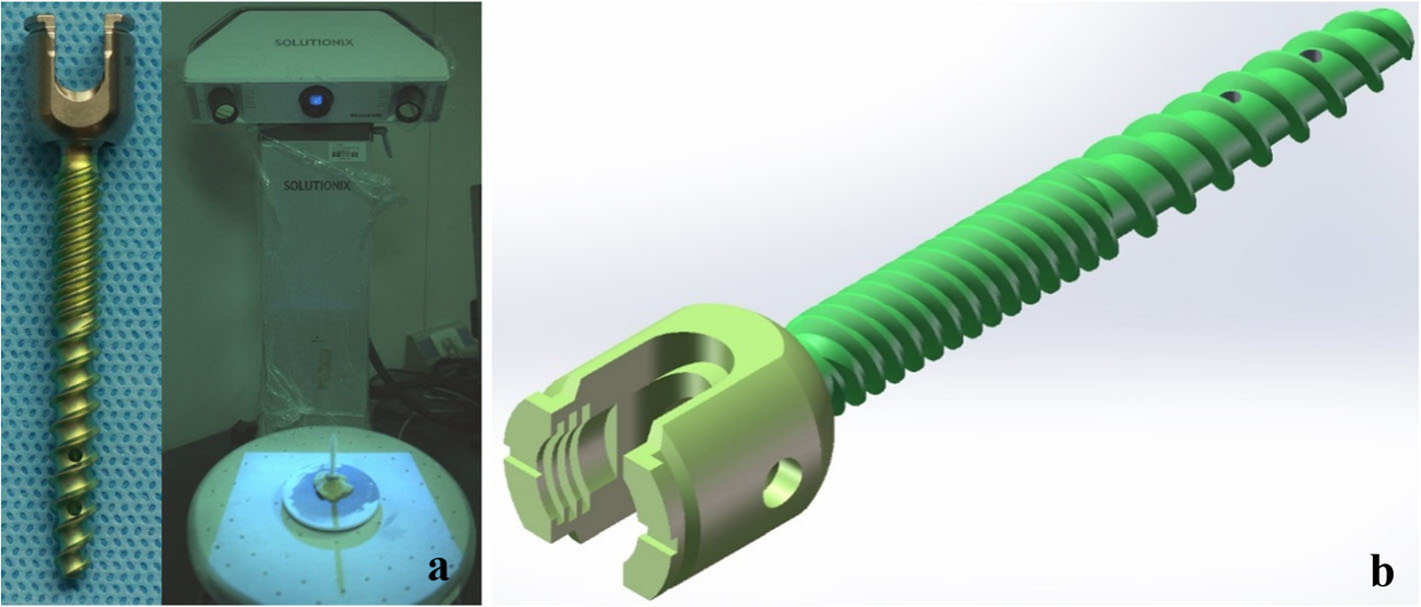
**a** The fenestrated pedicle screw material and picture of the 3D scanner working; **b** the model of the fenestrated pedicle screw

The 3D models generated by the scanner were imported into Geomagic Studio 2013 and SolidWorks 2017CAD for further processing. Finally, the models with realistic geometry were used for the assembly of surgical models. The length and outer diameter of the pedicle screws (DePuy Synthes, California, USA) were 50 and 6.0 mm, respectively (Fig. 1).

### The model of bone cement

By using a random number table of CAPSI patients, a patient who was undergone fenestrated pedicle screw with cement-augmented was randomly selected from the table. The cement model was constructed by using the postoperative lumbar CT data through the above software. The volume of bone cement was approximately 1.73cm^3^ and distributed in a lump pattern. Then, the cement model with 1.73 ml PMMA was scaled to 1 ml and 2.5 ml.

### Construction of instrument models with different volumes of PMMA

**Fig. 2:**
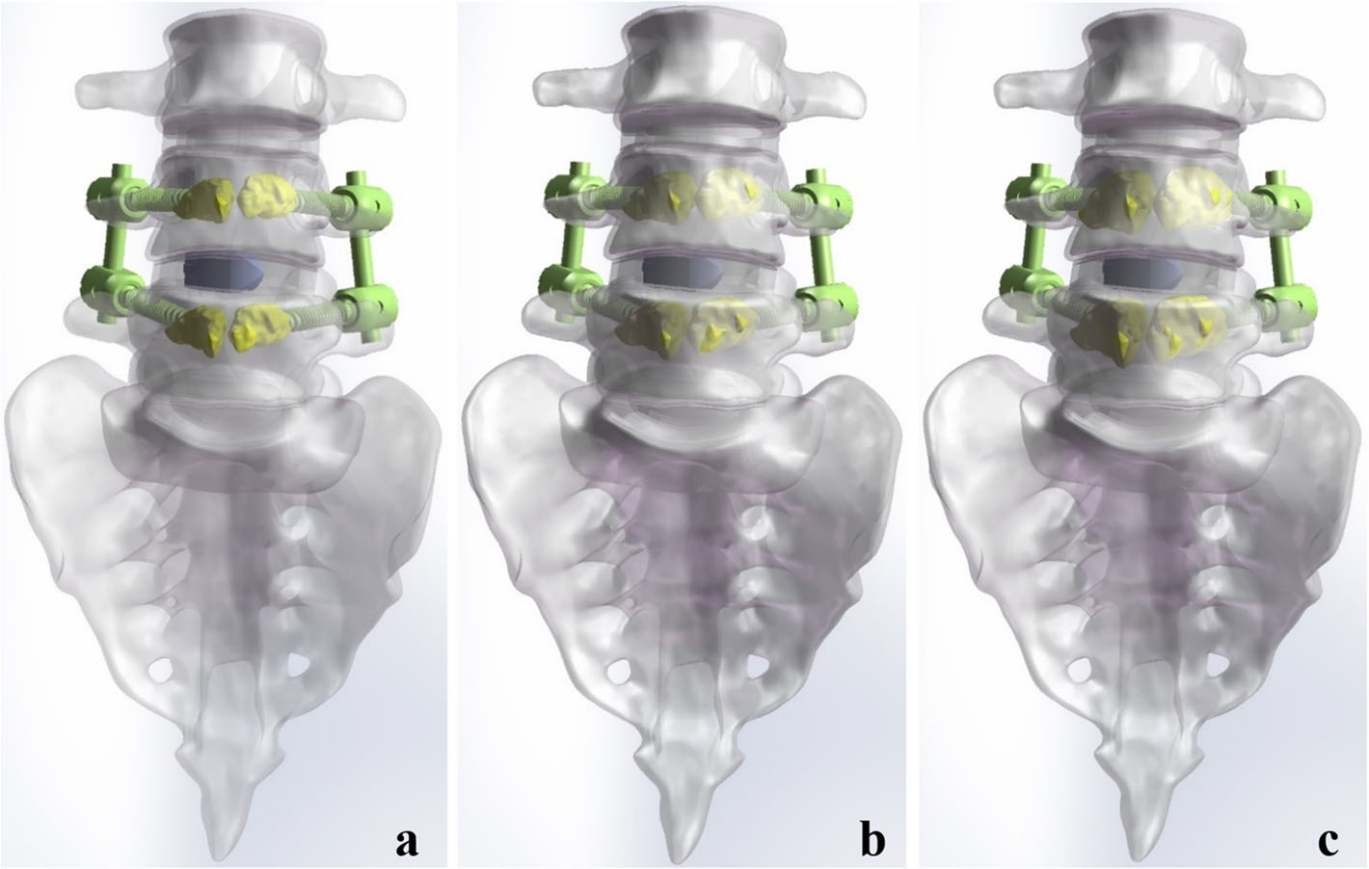
The models of CAPSI following single-level lumbar interbody fusion (**a**)1.0 ml PMMA per screw; **b** 1.73 ml PMMA per screw; **c** 2.5 ml PMMA per screw

**Fig. 3:**
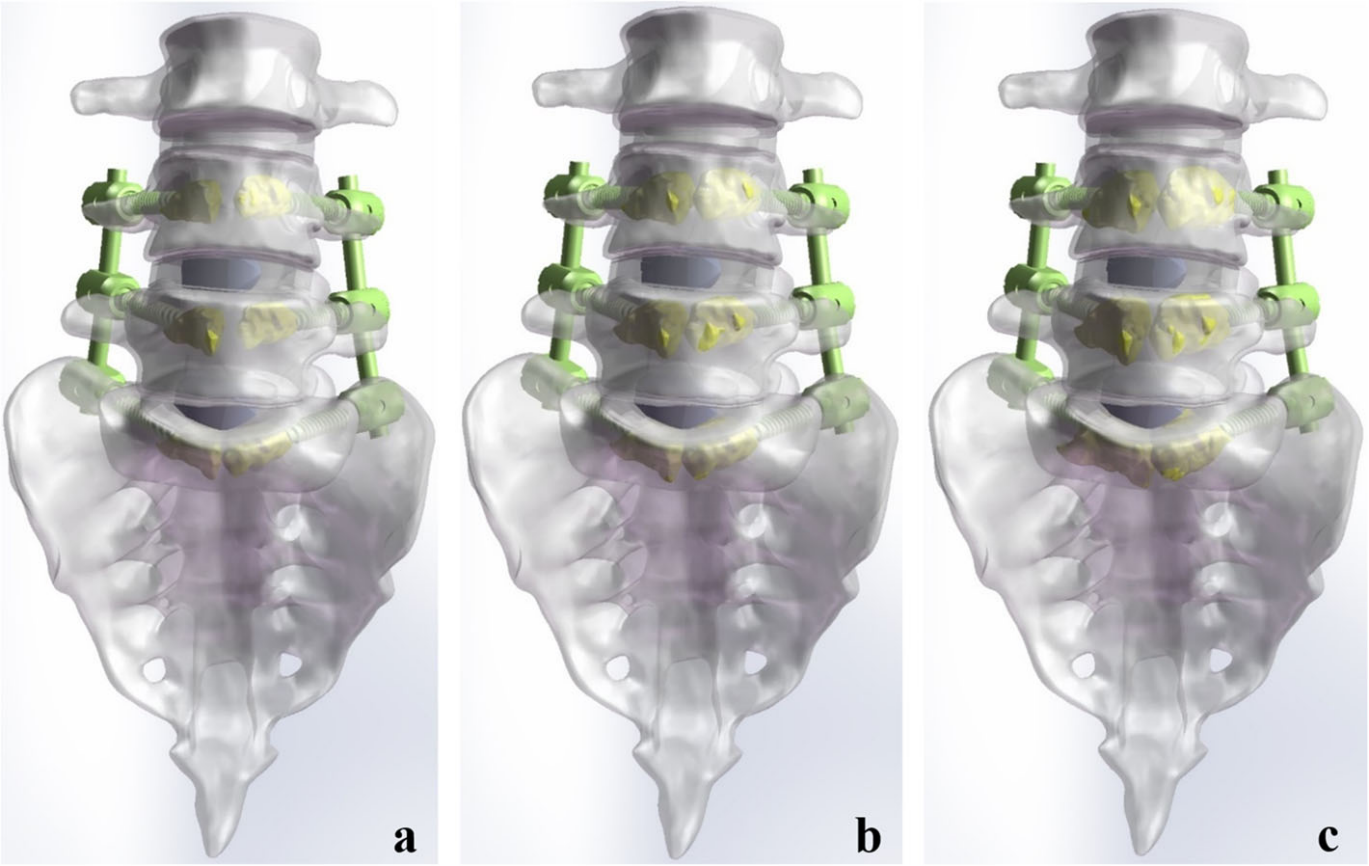
The models of CAPSI following double-level lumbar interbody fusion (**a**)1.0 ml PMMA per screw; **b** 1.73 ml PMMA per screw; **c** 2.5 ml PMMA per screw

The models of cage and rod were constructed in the SolidWorks 2017CAD according to the physical cage and rod. The outer diameter of the rod was 5.5 mm. The length and height of the cage were 24 and 12 mm. Subsequently, unilateral transforaminal lumbar interbody fusion (TLIF) was assumed in right to remove the facet joint, facet cartilage, part of the annulus fibrosus, cartilaginous endplate, and nucleus. The screws, cement, rod, and cage were integrated with the lumbosacral model to construct six surgical models. The interbody cage is placed in the center of the intervertebral space. To control variables and maintain consistency, the consistent location of cages, screws, rods, and bone cement were used in the different surgical models (Figs. 2 and 3).

### Loading and boundary conditions

All the FE model was imported into ANSYS Workbench 17.0 (ANSYS, Ltd., Canonsburg, Pennsylvania, USA) for biomechanical testing. The material properties of cortical bone (osteoporosis), cancellous bone (osteoporosis), endplates, nucleus pulposus, annulus fibrosus, facet cartilage, cages, bone cement, and posterior spinal instrumentation was set according to previous studies (Table 1) [10, 12, 13]. The ligaments of the spine were simulated using tension-only and nonlinear spring elements [14]. The contact type of the facet joint was defined as “frictional”, and the friction coefficient was set at 0.1. The remaining bodies were defined as the “bonded” mode [11]. To reach a more accurate calculation, the tetrahedron mesh was used and the character of mesh was set up according to previous reports: the dimension of the joint cartilage mesh was 0.5 mm, while that of the other bodies was 2.0 mm. Finally, the loading and boundary conditions of the six surgical models were set up [10, 13]: The sacroiliac joint was bilaterally fixed with all degrees of moment restricted throughout the whole analysis. a vertical compressive force of 150 N was used on the upper surface of L3, and a 10 Nm moment was applied along the radial direction in flexion, extension, left lateral bending, right lateral bending, left rotation, and right rotation. The ROM, the disc stress at L3–4, and inferior articular process stress at L3 were recorded to make a biomechanical comparison of different volumes of PMMA in adjacent segments.

**Table 1:** Material properties used in finite-element model

Material Properties	Young’s Modulus (E: MPa)	Poisson’s Ratio (μ)
Osteoporotic cortical bone	8040 (67% of normal)	0.3
Osteoporotic cancellous bone	34 (34% of normal)	0.2
Cartilage	50	0.3
Endplate	1000	0.3
Annulus fibrosus	4.2	0.45
Nucleus pulposus	1	0.499
Ligament		
Anterior longitudinal	20	0.3
Posterior longitudinal	20	0.3
Transverse	59	0.3
Ligamentum flavum	19.5	0.3
Interspinous	12	0.3
Supraspinous	15	0.3
Capsular ligament	7.5	0.3
Spinal instrumentation (titanium alloy)	110,000	0.28
Bone cement (PMMA)	3000	0.4
Spinal cage (polyetheretherketone)	3600	0.25

*PMMA* Polymethylmethacrylate

## Results

We compared our range of motion (ROM) results with those experimental data conducted by Yamamoto et al. [15]. The ROMs of different segments were in accordance with the previous literature under flexion-extension, lateral bending, and rotation loads (Table 2).

**Table 2:** Comparison of ROM between the intact model and the in vitro study at different levels

	Flexion	Extension	Bending	Rotation
L3-L4 (°)				
The intact model	6.66	5.41	5.33	1.64
Yamamoto et al	6.1	3.89	4.3	1.9
L4-L5 (°)				
The intact model	7.29	4.19	5.23	2.61
Yamamoto et al	7.1	4	4.1	1.8
L5-S1 (°)				
The intact model	7.35	3.95	3.43	1.86
Yamamoto et al	7	4.8	3.7	1.00

### Range of motion

**Fig. 4:**
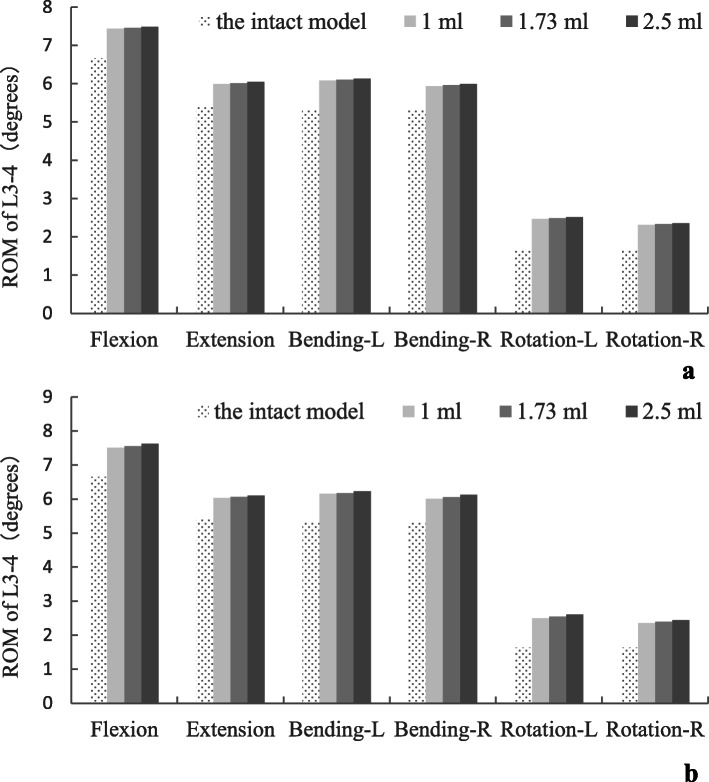
The ROM of adjacent segments following single (**a**) and double-level (**b**) lumbar spinal fusion

As shown in Fig. 4, the instability of the adjacent disc was accentuated by the CAPSI. The ROM at L3–4 increased in all motion cases to compensate for the reduction in the fixed segment. With the increased volume of PMMA, this effect was slightly magnified, reaching the highest ROM in flexion movement for both the single- and double-level lumbar interbody fusion models (Fig. 4). The variation of ROMs in the double-level lumbar interbody fusion models was more evident, particularly in rotation. The calculated data showed that the ROM at L3–4 was nearly unaffected by different volumes of PMMA in both single-level and double-level fusion models.

### Stress of the disc

**Fig. 5:**
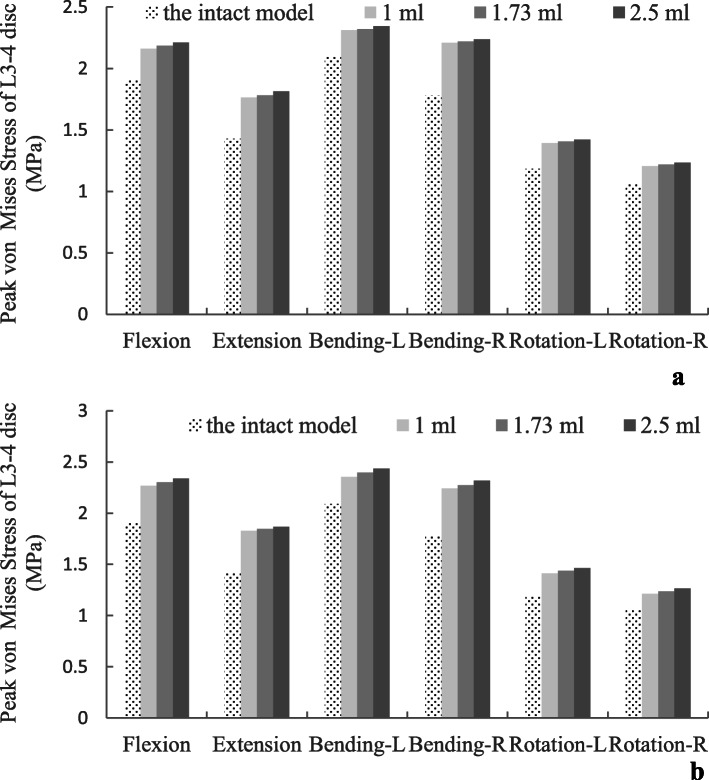
The disc stress of adjacent segments following single (**a**) and double-level (**b**) lumbar spinal fusion

**Fig. 6:**
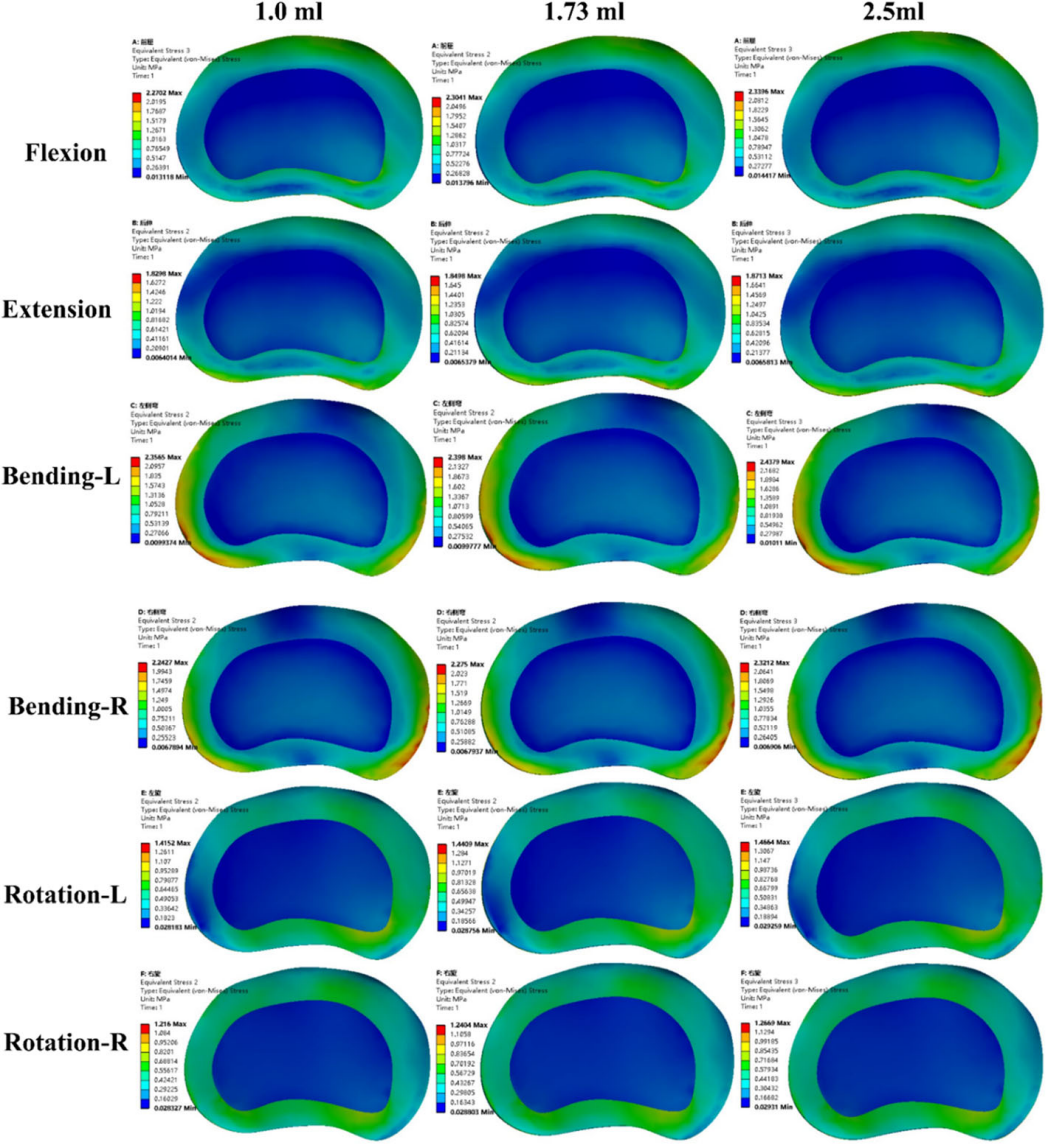
The peak von Mises stress distribution of L3–4 disc in double-level CAPSI model with different volumes of PMMA

Attending to the peak von Mises stress of the disc, compared with the surgical models, the disc stress for the intact model was still lower in all motion modes. Flexion and lateral bending movement were the worst motion modes in CAPSI models, as in the ROM analysis, for the upper adjacent disc. Although the disc stress increased in the CAPSI models under all loading conditions, the results were similar for different volumes of PMMA. The results showed that the PMMA volume did not have a significant effect on the adjacent disc (Fig. 5). The peak von Mises stress of the disc in the double-level CAPSI model with different volumes of PMMA (1, 1.73, and 2.5 ml) were calculated and are shown in Fig. 6.

### The stress of facet

**Fig. 7:**
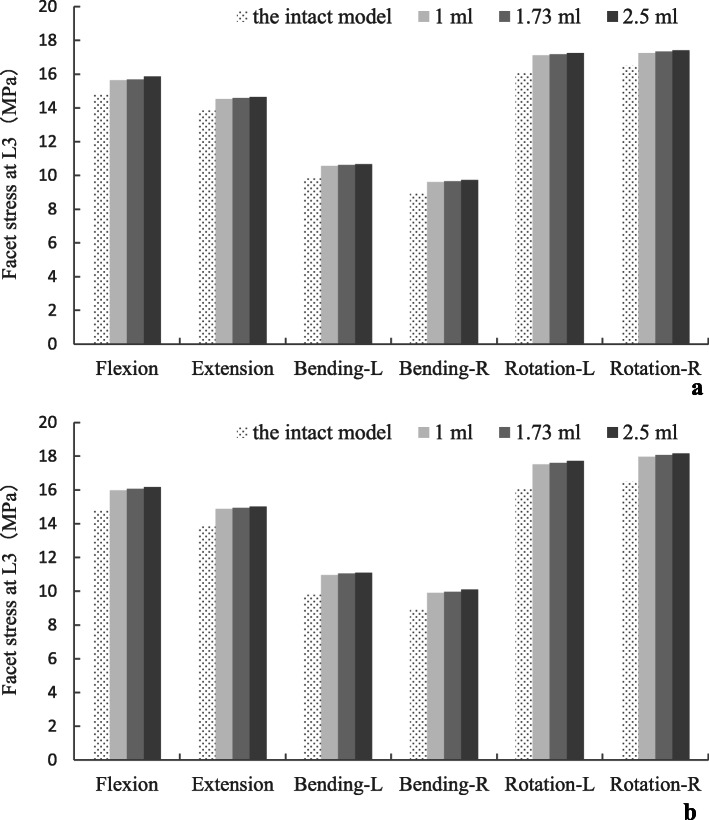
The facet stress of adjacent segments following single (**a**) and double-level (**b**) lumbar spinal fusion

The maximum stress in the inferior articular process (L3) is displayed in Fig. 7. The facet stress of surgical models was slightly higher than that of the intact model. Similarly, with a higher dose of injected PMMA, the facet stress was increased, while the gap among different models was still narrowed.

## Discussion

With advances in surgery and anesthesia, posterior lumbar fusion and pedicle screw instrumentation have been used progressively more often in aged patients with lumbar degenerative disease by spine surgeons. However, screw loosening, migration, and back-out is the most common postoperative complication of the pedicle screw, which usually results in painful nonunion, progressive kyphosis, and revision surgery [16]. Clinical studies have reported an overall instrumentation failure rate of 1 to 15% in ordinary patients, even 10 to 62.8% in patients with osteoporosis [17–20]. Reports in the surgical literature indicate that CAPSI has been widely used to enhance fixation strength to improve pedicle screw stability in osteoporotic spines [21]. However, previous reports also showed that a rigid instrument may grossly alter the physiologic load transmission at the instrumented level and has a cascading degenerative effect over the adjacent discs [22, 23].

It is generally agreed that ASD can be divided into radiologic adjacent segment degeneration (ASDeg) and adjacent segment disease (ASDis). Studies have documented a rate of clinical ASDis between 2 and 12.2% at different follow-up periods [24, 25]. For patients with ASDis who underwent revision, the satisfaction rate was approximately only 54%, which is significantly lower than that of other patients (83%) [26]. In addition, the initial disc degeneration of adjacent segments in elderly patients was more severe than that in younger patients, which has been reported to increase the incidence of ASD. Thus, for the study we report here, we planned to clarify the effect of different volumes of PMMA on ASD among aged patients and to provide a useful reference to spinal surgeons when considering CAPSI for early patients.

Finite element analysis can accurately characterize the complex biomechanical mechanism of the spine and clearly show the stress distribution of each part. Proper geometric characteristics of models are essential for the accurate outcome of FE analysis. In previous finite element studies, the three-dimensional solid models of the pedicle screw and bone cement were constructed by using SolidWorks or Hypermesh software [27, 28], which cannot accurately simulate the characteristics of the material object. Therefore, in the current study, the threaded pedicle screw (Fig. 1) and lumpy bone cement were constructed to be realistic with a 3D scanner and the patient’s CT data, respectively. In addition, lumbar degenerative diseases such as lumbar spinal stenosis occur mostly in the L4-S1 segment. Biomechanical experiments found that both solid and fenestrated screws can significantly increase the pull-out force in cement-augmented pedicle screw fixation [16]. And fenestrated screws were used more commonly in CAPSI. Thus, this study used a model of L4–5/ L4-S1 segmental fusion and fenestrated screws fixed to analyze the effect of the volume of PMMA after CAPSI.

In this study, to distinguish the influence in the adjacent discs, the ROM, disc stress, and facet stress were compared with the intact value. An increase in the ROM, disc stress, and facet stress were found in adjacent segments in all loading directions that were more pronounced in the double-level CAPSI model with 2.5 ml PMMA (the increasing stress on disc and facet may be related to the increasing segmental instability). However, the variation in single- and double-level CAPSI models was similar for various volumes of PMMA (1 ml, 1.73 ml, and 2.5 ml). Although CAPSI increases the risk of adjacent segment degeneration, this study did not find meaningful associations between ASD incidence and the volumes of PMMA. Otherwise, experimental data have reported that a cement volume between 1.0 and 3.0 ml significantly improves screw stability, whereas a volume beyond 3.0 ml does not increase the purchasing strength linearly but results in an increase of cement leakage [21, 29]. Therefore, within a certain range, increasing the volume of PMMA does not significantly affect the stability of adjacent segments, and PMMA volumes between 1.0 ml and 2.5 ml can be selectively used according to different degrees of osteoporosis.

This computational study was based on finite element analysis and has some limitations. First, because the scanning accuracy of the 3D scanner in the uneven and subtle parts of the screw (such as the screw thread) is insufficient, further processing is required in the scanned models by Geomagic Studio 2013 and SolidWorks software, and the extra processing may result in a modicum of distortion in the model. Furthermore, several simplifications were necessary for creating the finite element model, such as the characteristics of ligaments, paraspinal muscles, and body weight, which also limited the results. Otherwise, it is difficult to accurately simulate the interaction between trabecular bone and bone cement. Therefore, further cadaver studies and clinical observations are necessary to reach a more precise conclusion.

## Conclusion

The observed results suggested that CAPSI could increase the incidence of disk degeneration in the adjacent segment, while within a certain range, different volumes of PMMA provided an approximate impact over the adjacent segment degeneration. Clinically, PMMA volumes between 1.0 ml and 2.5 ml can be selectively used according to different degrees of osteoporosis.

## Data Availability

The datasets used and/or analyzed during the current study are available from the corresponding author upon reasonable request.

## Abbreviations

PMMA: Polymethylmethacrylate
CAPSI: Cement-augmented pedicle screw instrumentation
ASD: Adjacent segment degeneration
ROM: Range of motion
FEA: Finite element analysis
TLIF: Transforaminal lumbar interbody fusion
ASDis: Adjacent segment disease
